# Parent-child proximity and personality: basic human values and moving distance

**DOI:** 10.1186/s40359-016-0132-5

**Published:** 2016-05-17

**Authors:** Stefan Stieger, David Lewetz

**Affiliations:** Department of Basic Psychological Research and Research Methods, School of Psychology, University of Vienna, Vienna, Austria; Research Methods, Assessment, and iScience, Department of Psychology, University of Konstanz, Konstanz, Germany

**Keywords:** Basic human values, Parent-child proximity, Value theory, Dominance analysis

## Abstract

**Background:**

An important event in many young people’s lives is moving out of the parental home. This event is often operationalized as the distance between parents and their children, i.e., parent-child proximity.

**Methods:**

The present study (*N* = 1,451) analyzed correlates of parent-child proximity through the lens of human value theory (Schwartz, Advances in experimental social psychology, 1992). Besides a classical proximity measure (i.e., parent-child), we also calculated the distance between childhood and current place of residence (i.e., childhood-now), as well as parent-childhood proximity (distance between children’s childhood place of residence and the current place of residence of parents), which acts as a control group because this distance is most probably chosen by the parents.

**Results:**

As hypothesized, we found that participants valuing universalism and self-direction as important (i.e., associated with growth and anxiety-freedom) moved further away from the place where their parents live and the place where they grew up than participants valuing self-protection and anxiety-avoidance (e.g., tradition, security, conformity).

**Conclusions:**

This study not only adds to research on psychological motivations to move, it endorses value theory as being a useful lens through which to analyze migration behavior.

## Background

Migration – stimulated by globalization – is increasingly emerging as an important sphere of societal and civic interest. But moving from one place to another has always naturally occurred, typified by when children leave their homes for work or to set up families of their own [27]. This behavior has fallen under the umbrella topic of parent-child proximity (e.g., [15, 19, 23, 39]).

Research about parent-child proximity has analyzed sociocultural aspects, such as through an examination of family bonds that are assumed to be tighter in southern regions of Europe than northern regions (e.g., [29]). Moreover, demographic aspects, such as sex, age, marital status, education, or family size (for a discussion, see [16]), as well as socioeconomic aspects such as financial support through the family [39] and geographical aspects such as the attractiveness of places [1], have all been frequently studied. Besides these sociological, demographic, economic, and environmental studies, psychological research has also examined determinants of the decision to change one’s residence.

For example, Jokela et al. [18] analyzed temperament traits (i.e., emotionality, sociability, and activity) in a large prospective study in Finland. They found that more (vs. less) sociable individuals had greater moving distances and were more likely to move to urban (vs. rural) areas. Furthermore, individuals high (vs. low) in the temperament trait of emotionality had decreased moving distance, but an increased likelihood of leaving the parental home. The Big Five personality traits have also been analyzed in terms of moving behaviors. It has been found that men (but not women) higher in neuroticism and extraversion were more likely to move [35]. In short, past research has found that moving decisions are not only influenced by demographic aspects (e.g., education), sociological aspects (e.g., family bonds), genetic dispositions [8], and economic considerations (e.g., earnings), but also by psychological aspects such as temperament traits, personality, and affect [37].

But what is the motivation to move? Another potentially interesting lens that could guide moving behavior is values. Values are considered as being guiding principles and motivations in human life, feeding our goals [31]. Perhaps the most influential theory about values is ‘personal value theory’ [31], which proposes a widely-accepted, fine-grained model of basic human values. The level of detail of this model makes it ideal to analyses motivations to move.

### Basic human values

**Fig. 1:**
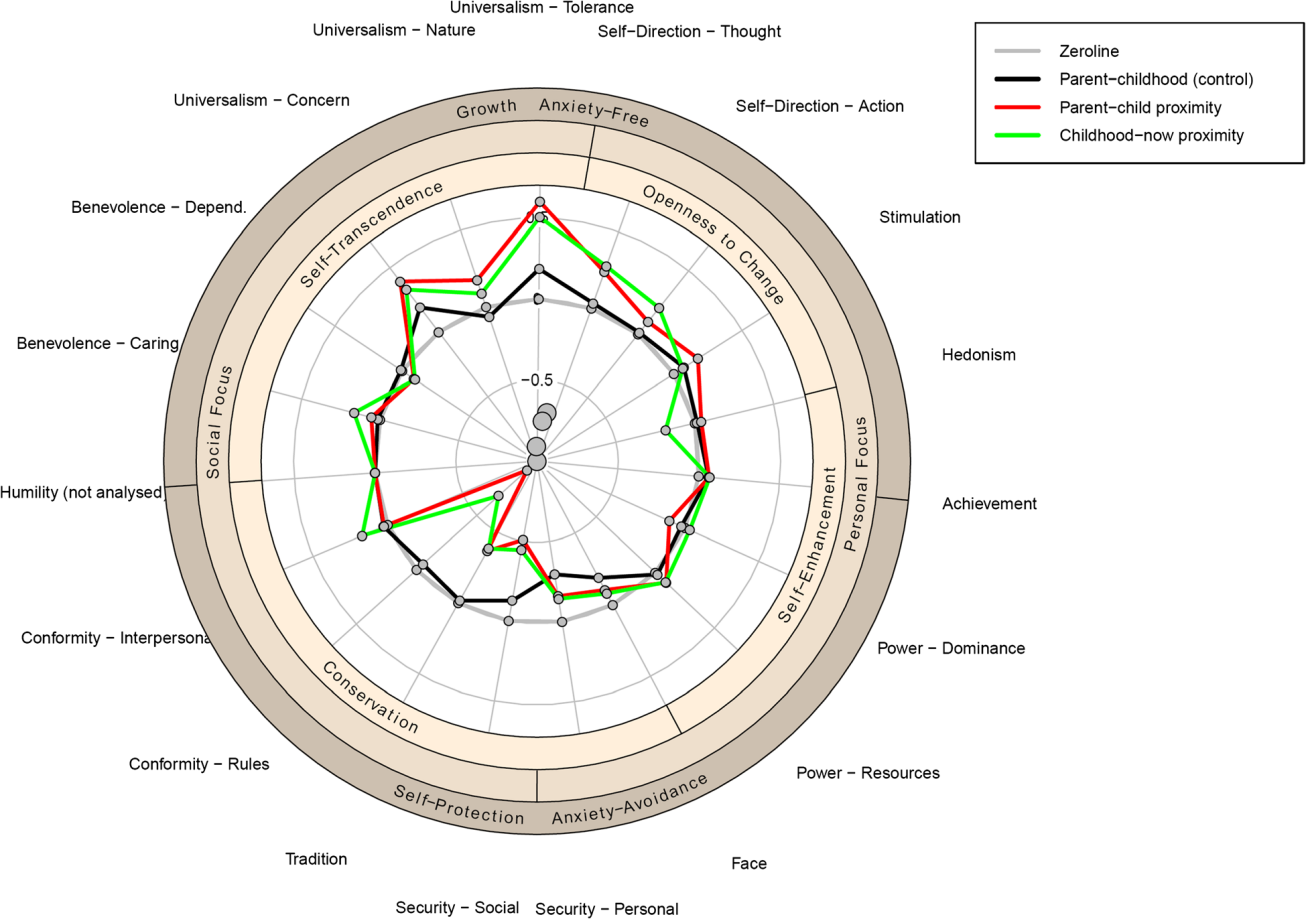
Radial plot of the explained variance of all 19 values onto migration distance. Explained variance values in % from the dominance analysis. Direction of the effects based on the sign of the Spearman correlation coefficient. Humility was excluded by setting the values to zero

The concept of values has a long history (e.g., [14]) and, largely because of its universal nature in relation to human behavior, has emerged as an important concept in several scientific disciplines, such as psychology, sociology, business, management, and politics (e.g., [33]). In particular, Schwartz [31] proposed a widely-accepted model of basic human values based on 6 main features: values are beliefs, values are desirable goals, values transcend specific actions and situations, values serve as standards or criteria, values are ordered by importance, and the relative importance of multiple values guides action (for a detailed description, see [34]). Based on these main features, Schwartz described ten broad values that he assumed to be universal across humans and cultures [5, 13, 32]. These values can be described as a guiding standard of human life, which are fundamentally important and a central part of identity. However, the values are not independent from each other; rather, they have dynamic relationships with each other and can be organized in a circular structure (circumplex model: [31]; see also Fig. 1). Values close to each other regarding their circular configuration share some congruency in their underlying motivation, whereas values opposite to each other are in conflict. For example, Hedonism is close to Stimulation in the circular order, which is reflected by a positive correlation, whereas Hedonism is opposite to Humility in the circular model and is therefore negatively correlated with this value. Based on this view, further higher-order factors can be described on bipolar dimensions that form a continuum of related motivations (e.g., self-transcendence vs. self-enhancement; conservation vs. openness to change; see [34]). This structure has been confirmed in several cross-cultural studies (e.g., [5]). It is important to note that it is assumed that cultures do not differ in the structure of basic values rather than in the importance they attribute to the respective values. Meanwhile, an even more elaborate model has been proposed by Schwartz, in which 19 different values are differentiated ([34]; see also [9]).

In short, Schwartz’s [31] value theory attempts to explain human action on the same fundamental level as personality theories (e.g., Big Five). Furthermore, human values are multi-faceted, but can condensed to broad basic motivations (e.g., growth vs. self-protection; see Fig. 1, outer circles). This makes them ideal to analyze moving behavior because moving itself is influenced by many diverse decisions, not only sociocultural and socioeconomic needs, but also psychological ones, such as certain personality constellations [42] or affect [37]. Furthermore, one main feature of basic human values is that the relative importance of multiple values guides action, i.e., moving behavior is probably not guided by one single value, but rather by the interplay of several values.

### Research question

Data collection for the present study took place in German-speaking countries of Europe, which represent long-term politically stable, economically-developed societies with a high standard of living. Therefore, we can expect that participants were generally motivated to move by an optimistic outlook in life, rather than a pessimistic one as proposed by deficiency models [45]. Deficiency models postulate that a lack of personal and social resources are the driving forces behind moving, at least in countries with political and/or economic problems. This is also in line with results of Stieger et al. [37], who found that parent-child proximity was associated with positive affect, where affect can be best described as a sort of basic mood, which in turn is the breeding ground for emotions. Individuals with high positive affect (i.e., enthusiastic, active, and attentive; [44]) moved further away from their parents than individuals with low positive affect.

Research question 1: If value theory is a useful lens through which to analyze migration behavior, then according to the deficiency model German-speaking participants should be generally motivated to move because of an optimistic outlook in life. If this is the case, then values associated with growth and anxiety-freedom should be positively associated with parent-child proximity, whereas values associated with self-protection and anxiety avoidance should be negatively correlated with parent-child proximity.

When it comes to basic human values, Tartakovsky and Schwartz [38] attempted to integrate optimistic and pessimistic motives by suggesting a typology of potential motivations to move and related them to basic human values. They postulated four different potential motivations, each (except idealism, which was not assessed) expressed a set of basic human values. The preservation motivation (physical, social, and psychological security for oneself and one’s family) is associated with the higher order value of Conservation (Conformity, Tradition, and Security). Self-development (personal growth, acquiring new knowledge and skills) is associated with the higher order value of Openness to change (Self-direction, Stimulation, and Hedonism). Materialism (financial well-being, wealth, and control over material resources) is associated with the higher order value of Self-enhancement (Achievement, Power). Finally, idealism (building a better society) is associated with the higher order value of Self-transcendence.

This typology is also relevant for the present study. For example, individuals with high preservation motivations (i.e., reflected by the values of Tradition, Security, and Conformity) will be unlikely to move very far, as compared with individuals with a low priority for preservation (for a similar reasoning about identification with a nation, see [30]). Similarly, traditions are socialized during childhood and youth. Therefore, individuals who value tradition will be unlikely to move very far away from their parents’ place (or place of childhood), as compared with individuals who do not foster traditions. The same rational applies to security: Individuals who value safety and stability in society will be unlikely to move far away from the environment they live in (e.g., place were they grew up or place were their parents live), as compared with individuals who do not value safety. Similar arguments can be applied to all other values, as well as basic motivations formulated in the typology of Tartakovsky and Schwartz [38].

Research question 2: If value theory is a useful lens through which to analyze migration behavior, then according to the motivation typology of Tartakovsky and Schwartz [38], German-speaking participants who move very far away from their parents’ homes should be motivated by personal growth and acquiring new knowledge and skills, rather than physical, social, and psychological security for oneself and one’s family. If this is the case, then we would expect values associated with growth and anxiety-freedom to be more important than values associated with self-protection and anxiety-avoidance (see the three circular areas in Fig. 1), the further someone moves away from his/her familiar place of living (childhood, parents’ place).

## Method

### Power analysis

Research about moving behavior starts from the premise that this behavior is multicausal. This is also reflected by finding relatively weak effects and low explained variance values, because one study usually cannot address all possible predictors (e.g., [21]: *R*^2^ ~ 13 %; [36]: *R*^2^ ~ 2 %). One of the strongest predictors seems to be the educational level (‘brain drain’ hypothesis; e.g., β ~ .30 in [37]; see also [21]), but most significant predictors are of relatively weak effect size. Therefore, we assumed we would likewise find weak effects in the present study (*r* = .1 according to [10]).

An as yet overlooked aspect regarding power is the reliability of the measures used. If measures are unreliable, then power is also reduced (for a discussion, see [20]). Therefore, we also accounted for lower measurement reliability. The lowest presented measurement reliability presented in Schwartz et al. [34] was .63, which was found for the Humility value. Based on this lowest measurement reliability value, we calculated a corrected lower effect size of *r* = .079 as the basis for the power analysis (for calculation details, see [17]; for a discussion, see [6]). Because we were interested in the predictive value of each single predictor and not all predictors together, we chose the bivariate normal model for correlations instead of the linear multiple regression model. Based on this analysis, the new minimum sample size to detect effects was *N* = 1,255.

### Participants

The recruited sample size was larger than the one required, which should additionally benefit statistical power (required: *N* = 1,255; recruited: *N* = 1,450). Participants (54 % women) were German-speaking volunteers (*M*_age_ = 44.2 years, *SD* = 16.2; range 18 to 99 years) recruited by word-of-mouth through friends and relatives of several research assistants, constituting a convenience sample. We used six different age-strata (18 – 25, 26 – 30, 31 – 40, 41 – 50, 51 – 60, 61+) with the aim of an equal number of participants in each strata in the final sample by using a systematic sampling approach (i.e., first, strata are filled up by random sampling; if a strata is full, then the remaining strata are filled up by systematic sampling). This ensured a broad range of participants who already had their own households (i.e., had moved away from their parents’ home).

In terms of educational qualifications, 10 % had completed primary education, 32 % had an apprenticeship diploma, 33 % had completed secondary education, and 25 % had a university degree. Participants’ current relationship status was: 16 % single, 26 % in a relationship, 51 % married, 4 % divorced, and 3 % widowed.

### Materials

#### Portray value questionnaire-revised (PVQ-R)

The PVQ-R [34] is a 57-item measure to assess 19 different human values (see Fig. 1). Each item presents a fictitious person’s goals, aspirations, or wishes that point to a particular value. Participants were asked to state how strongly they identified with the particular portrayed person on a 6-point Likert-type scale (1 = not like me at all, 2 = not like me, 3 = a little like me, 4 = somewhat like me, 5 = like me, 6 = very much like me). Internal consistencies were mostly acceptable for 3-item scales (see Table 2; range .56 to .88) and comparable with past research ([34]; range .63 to .85), except for the value Humility, which was below .50 in our study (.476). Therefore, we have excluded Humility from all further analyses. Value scores were ipsatized prior to analysis, i.e., participant’s mean score across all 57 items were subtracted from the value score of each value item. This follows standard procedure (e.g., [3, 31]) and is done in order to control for individual response tendencies, which could create random variability, and also because it is the relative importance of a value compared to other values that matters, rather than the importance of a value per se.

#### Place of residence

Research on determinants or correlates of moving behavior (e.g., sex, age, marital status, education, family size, attractiveness of places), operationalized as distance between two residences (e.g., parent-child proximity), is characterized by low explained variance values (e.g., [21]: *R*^2^ ~ 13 %; [37]: *R*^2^ ~ 10 %, range of significant beta weights between .05 to .31). This may be explained as a function of several issues. First, moving is grounded in many (cultural, economic, demographic) aspects and, therefore, will be unlikely to be predicted by a single factor, but rather by many factors that each explain a small amount of the variance. Second, the measurement of distance between two places is not error-free. For example, almost all studies using distances do not use the real distance between two addresses (usually because of privacy concerns), but rather geographical positions of the center of postal code areas or municipalities. Furthermore, there is some debate as to whether the real distance (e.g., when driving on the road), the distance as the crow flies (i.e., linear distance between two places), or even travel time is the best operationalization of migration. Third, people often live in several places. For example, many students live in student halls near their homes and some people have secondary residences for leisure or work reasons (e.g., because of large commuting distances). Thus, it is often difficult to clearly define a certain place (e.g., the place ‘home’).

To keep the measurement of distances as error-free as possible, we took the following considerations into account. First, we tried to assess the distance as accurately as possible. Asking participants about their real address violates ethics standards concerning anonymity. Therefore, we used the postal code area, which is not as precise as the real address but more precise than, for example, the municipality. Second, we calculated three operationalizations of distance: the real distance when driving on the road, the distance as the crow flies, and the travel time. Third, when measuring distances, there was the question of the geographical reference point. Past research has used several approaches, including the last residence, the place of birth (e.g., [18, 43]), the place where the parents live (parent-child proximity: e.g., [22]), and so forth. Therefore, we calculated three different measures of distance:

1. We used the current place of parental residence to assess parent-child proximity, as has been done frequently in past research
2. Because the distance between parents and their (adult) children is also influenced by the moving behavior of the parents after children have left the home, we also assessed the place of childhood where participants predominantly grew up (until ~ 10 years of age) to calculate a childhood-now proximity. We assumed that the decision to move was made by parents when children were young; after that, this decision is more often determined by the children themselves as they age (e.g., getting a first job, raising their own family). Therefore, the childhood-now proximity could be a more valid measure of moving than the parent-child proximity. Furthermore, this second measure of proximity enabled us to conduct sensitivity analysis [28] to show whether the results hold stable when using a slightly different measure.
3. Finally, we calculated the parent-childhood proximity, which acts as a control condition. The children’s childhood place of living is the place when the family was still young (i.e., parents live together with their young children). This place was most probably chosen by the parents. The current place of parents was also chosen by the parents. Therefore, the parent-childhood proximity should be largely independent from influences of parent’s children.

To calculate these distances, participants were asked about their current place of residence (country, place, postal code), which they would describe as ‘home’ following the place attachment concept (i.e., emotional bond between a person and a particular place; [1, 24]). Furthermore, participants were asked about their mothers’ and fathers’ current (or if already deceased, the last) place of residence (country, place, postal code) to calculate the current parent-child proximity, as well as the place of childhood where participants predominantly grew up (until ~ 10 years of age).

### Procedure

Participants gave their informed consent, completed the PVQ-R along with several other measures that were not part of this study, and finally provided demographic details (age, sex, highest educational level, current relationship status, and places of residence). For the purposes of anonymity, each questionnaire was put into an envelope and thrown into a box. Furthermore, all participants took part on a voluntary basis and were not remunerated for participation.

### Analysis of Distances

In European German-speaking countries, municipalities (equivalent to US counties), are divided into several postal code areas. This comes with the advantage that postal code areas are geographically more precise than the municipality itself. Although the exact postal address would have been best for calculating the parent-child proximity (but problematic because of anonymity and ethics reasons), using distances between the centers of postal code areas seem to be a very good estimator of the real distance.

We determined three measures of proximity using the Google Geocoder API: the real distance between parents and their (adult) child using roads, the distance as the crow flies, and the journey time. The Google Geocoder API comes with the advantage of allowing for checks on postal codes and places for their validity. By applying a multistage approach, we first checked postal codes and places for their validity. Then, longitude and latitude coordinates were determined and proximities of two postal code areas were calculated, again using the Geocoder API. In the case of unclear postal codes or places, further information was used to clarify the issue (e.g., other stated postal codes or participants’ demographic data). If still unclear, postal codes and places were deleted for data quality reasons.

The road distance and journey time were automatically estimated from the optimal route, which was provided by the Google Geocoder API. All three measures have their advantages and disadvantages. The journey time might be more familiar to everyday experience (see also [41]) but has the disadvantage that journey time is also influenced by driving behavior and chosen route. The road distance has more face validity than the distance as the crow flies (e.g., in mountainous regions). Nevertheless, all three measures were highly correlated (all Spearman rank-order correlations *r*_sp_ > .99); we, therefore, decided to use the distance as the crow flies, which is common practice in research on proximity (e.g., [18, 37]).

**Fig. 2:**
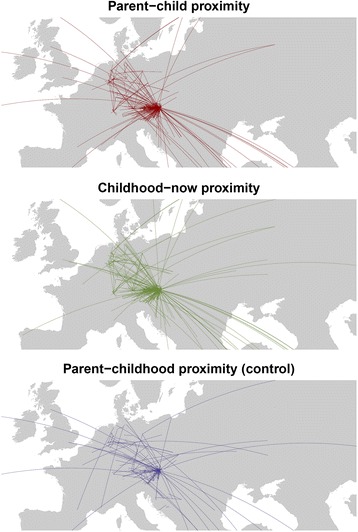
Geographical distances between parents and their (adult) children’s current place of residence, as well as distances between (adult) children’s current place of residence and the place where they grew up (i.e., place of childhood)

Distances were highly skewed (skewness > 6.3). Therefore, following standard practices, we log-transformed all distances (1 + log_10_) before further analyses (e.g., [18]).^1^ For a graphical overview of the distances between participants and their parents, see Fig. 2 (for ease of use, we plotted only the postal codes of mothers).

### Dominance analysis

Assessing a multicausal psychological phenomenon with expected low effect sizes comes with several problems. Using a multiple linear regression to assess the predictive value of several variables on the outcome measure proceeds on the assumption that multicolinearity is either very low or absent. Multicolinearity is prevalent when predictors share some variance (i.e., are intercorrelated). To control for multicolinearity, statistical packages calculate the so-called Variance Inflating Factors (VIFs) to evaluate this problem.

In the present study, multicolinearity was a particular problem for several reasons. First, the greater the number of predictor variables, the higher the probability of intercorrelations and the more complex is their interplay. Second, in contrast to the Big Five personality traits, the postulated 19 basic human values are assumed to correlate with each other. The closer the values in the circumplex circle (see Fig. 1), the higher their intercorrelations. Opposing values in the circumplex circle have negative correlations. Therefore, multicolinearity is expected. Third, multicolinearity is even more of a problem when effect sizes are expectably low. Low VIFs suggest weak intercorrelations but weak intercorrelations can have a substantial influence on estimating regression coefficients when the expected effect size (i.e., regression coefficient) is also low. Therefore, applying oft-articulated standards about acceptable VIFs (e.g., values above 10 are problematic; for a discussion, see [26]) cannot be applied.

To account for all these aspects, we decided to conduct a dominance analysis [2, 7]. Dominance analyses have the advantage of assessing the importance of each predictor relative to the other predictors in the model. This is done by looking at the contribution of a predictor in the linear model not only in conjunction with the other predictors, but also in isolation. Practically, all possible combinations of the predictor variables are used to calculate partial, direct, and total effect parts by decomposing the total *R*^2^ (explained variance). The partial effects are the contribution of all possible combinations of predictors on the outcome measure by excluding either one or more predictors from the model. The direct effect is the independent contribution without the other predictors in the model (i.e., zero-order correlation with the outcome measure). The total effect represents the contribution when all predictors are included in the model at once (i.e., the classical multiple linear regression). The outcome of the dominance analysis is composed of *R*^2^ values for each predictor representing the real explained variance (i.e., adjusted for shared variances with other predictors). In the present study, dominance analyses were calculated using the R package ‘yhat’ [25].

## Results

### Descriptives

In 37 % of cases, participants lived in the same postal code area as either their fathers, their mothers, or both parents. Furthermore, in 70 % of cases, the postal code of parents was identical (i.e., were probably still married or still lived together). In general, participants’ postal codes were widespread, resulting in 357 different postal codes. For those who had moved, distances to fathers were slightly smaller than to mothers (*Md*_mother_ = 38.4 km, *Md*_father_ = 38.0 km). For further descriptives of distances, see Table 1. For an overview of the geographical spread of all distances, see Fig. 2. Because father-child and mother-child proximities were highly correlated (*r*_sp_ = .91, *p* < .001), we used mean distance scores for further analyses (skewness = −0.5; *SE* = 0.07).

**Table 1:** Descriptives of distances in kilometer

Proximity	Definition	*n*	*Md*	*M*	*SD*	min	max
Parent-child proximity	Distance between parents’ and their (adult) children’s current place of residence.	1,364	8.9	153.7	597.7	0	9,825.0
Childhood-now proximity	Distance between participants’ place of childhood and their current place of residence.	1,444	8.0	122.1	350.0	0	4,555.8
Parent-childhood proximity (control)	Distance between parents’ current place of residence and their children’s place of childhood.	1,361	0.0	78.3	403.4	0	6,655.4

Distances are not log-transformed for descriptive purposes

In 32 % of cases, the parents’ place of residence was different from the (adult) children’s place of childhood, i.e., parents moved away from the place where their child grew up (see Fig. 2, third panel). This underlines our rational that using the parent’s current place of living might be not an optimal reference point of migration behavior.

The sample represents classical moving behavior within a particular culture (i.e., German-speaking European countries). The parents’ current place of residence was outside this cultural region (i.e., participants immigrated) in only 4.9 % of cases. Furthermore, the participant’s place of childhood was not in the German-speaking cultural region in only 5.9 % of cases.

### Dominance analysis

First, multiple linear regressions were calculated with all participants (i.e., including those with distance 0) for power reasons. For parent-child proximity and childhood-now proximity, significant predictors were found with explained variance values (*R*^2^) of 8.2 and 7.0 % respectively (see Table 2). As hypothesized, the multiple linear regression for parent-childhood proximity was not significant, with only 1.8 % explained variance. All VIFs, which are indicators of multicolinearity, were < 4. Following current practices, VIFs higher than 10 are regarded as problematic. As outlined above, this depends on the expected effect size (i.e., beta weights). The lower the expected effect size, the more a low VIF level should be regarded as problematic. In the current case, the impact of multicolinearity on the beta-weights could also be tested by calculating Spearman rank-order correlations between dominance weights (which should be true values adjusted for intercorrelations) and the absolute values of beta weights. If there is no multicolinearity, then the order ranks of dominance weights should resemble the order ranks of beta weights resulting in a perfect rank-order correlation of 1. The more the rank-order correlation deviates from this perfect correlation, the more likely it is that multicolinearity is a limiting factor. In fact, all three rank-order correlations were below 1 (parent-child proximity: *r*_sp_ = .54, *p* = .01; childhood-now proximity: *r*_sp_ = .51, *p* = .02; parent-childhood proximity: *r*_sp_ = .80, *p* < .001). Therefore, the dominance weights should be given preference over beta weights.

**Table 2:** Predictors of parent-child, childhood-now, and parent-childhood proximity

		Parent-child proximity			Childhood-now proximity			Parent-childhood proximity (control)		
		Dominance %	β	*r*_sp_	Dominance %	β	*r*_sp_	Dominance %	β	*r*_sp_
Demographics										
Age		0.23	−.022	−.098^a^	0.14	−.016	−.090^a^	0.25	−.041	−.071
Sex		0.90	.102^a^	.088^a^	0.65	.082^a^	.082^a^	<0.01	.003	.004
Education		2.70	.161^a^	.232^a^	2.18	.141^a^	.219^a^	0.12	.024	.058
Human values (PVQ-R)	Cronbach α									
Achievement	.668	0.06	.018	.037	0.07	.033	.029	0.06	.019	.026
Benevolence-caring	.732	0.05	.021	.030	0.17	.053	.047	0.02	−.003	.027
Benevolence-dependability	.693	0.09	−.037	−.008	0.10	−.039	−.004	0.01	−.007	.018
Conformity-interpersonal	.650	0.02	.010	.008	0.17	.055	.030	0.03	.005	.015
Conformity-rules	.880	0.92	−.079	−.163^a^	0.68	−.062	−.132^a^	0.05	−.022	−.040
Face	.647	0.10	.049	−.014	0.08	.046	−.010	0.19	−.046	−.049
Hedonism	.772	0.04	.003	.035	0.19	−.035	−.010	0.01	−.018	.022
Humility	.476 ^b^									
Power-dominance	.704	0.11	−.046	−.011	0.03	−.004	.012	0.03	−.030	−.011
Power-resources	.801	0.09	.051	.008	0.09	.056	.013	0.02	.007	.003
Self-direction-autonomy of action	.566	0.10	.010	.082^a^	0.21	.040	.092^a^	0.01	−.005	.025
Self-direction-autonomy of thought	.558	0.24	−.002	.118^a^	0.27	.019	.120^a^	0.03	−.023	.044
Security-personal	.778	0.16	−.008	−.095^a^	0.14	.003	−.094^a^	0.30	−.050	−.073
Security-societal	.795	0.51	−.018	−.147^a^	0.44	−.008	−.147^a^	0.13	−.020	−.065
Stimulation	.577	0.18	.021	.111^a^	0.06	.006	.090^a^	0.07	.009	.044
Tradition	.855	0.37	−.025	−.115^a^	0.38	−.020	−.115^a^	0.02	<.001	−.028
Universalism-concern	.761	0.39	.044	.099^a^	0.33	.044	.100^a^	0.19	.037	.062
Universalism-nature	.858	0.18	.050	.035	0.09	.043	.013	0.06	−.036	−.014
Universalism-tolerance	.736	0.60	.063	.118^a^	0.50	.064	.106^a^	0.19	.021	.071
		F(21,1341) = 5.67,			F(21,1422) = 5.12,			F(21,1338) = 1.17,		
		*p* < .001; *R* ^2^ = 8.2 %			*p* < .001; *R* ^2^ = 7.0 %			*p* = .272; *R* ^2^ = 1.8 %		

Coding of Sex: 1 = men; 2 = women. *r*_sp_ = Spearman rank-order correlation. ^a^significant after correction for false discovery rates due to multiple testing [4]*.* ^b^ Due to the low reliablity, Humility was excluded from all analyses

As expected, demographic variables, such as participant’s sex, age, and highest educational level, explained large amounts of the overall variance (3.8 % and 3.0 % respectively), but only for the parent-child and childhood-now proximity (parent-childhood proximity: 0.4 %). Women moved farther away from their parents and their place of childhood than men (distance to parents: *Md*_men_ = 33.2 km, *Md*_women_ = 42.1 km; distance to childhood place: *Md*_men_ = 34.7 km, *M*_women_ = 42.5 km). Furthermore, the higher the educational level, the higher was the proximity to parents and childhood place (average ∆*Md* gain of 25.3 km and 21.0 km, respectively, for each educational level). Education was the strongest predictor in line with past research (see also [21]).

Interestingly, the relative importance of basic human values explained a similar amount of variance in the outcome measure as compared with demographics. The sum of all dominance weights from all 19 values explained 4.2 % for parent-child proximity and 4.0 % for childhood-now proximity (parent-childhood proximity: 1.4 %). More specifically, the farther the distance the lower was the importance for values associated with the higher order value of Conservation (e.g., Security-social, Tradition, Conformity-rules), but the greater was the importance for values of the higher order value of Self-transcendence (e.g., Universalism-concern, Universalism-tolerance) and Openness to change (e.g., Self-direction). This is in line with the assumption that values opposite to each other in the circumplex model (see Fig. 1) should be antagonistic, e.g., the higher the importance of growth values, the lower should be the importance of self-protection values.

Figure 1 shows the circular structure of all 19 basic human values together with the higher order values in the outer circles. Values represent the dominance weights (see Table 2) and the direction of the dominance weights is based on the sign of the zero-order Spearman rank-order correlation. Furthermore, Fig. 1 shows centroids, which are the mean of the respective *x* and *y* values. These centroids have to be interpreted with caution because the circumplex structure of basic human values does not imply that all intervals are equal (i.e., values all of the same circular distance to the neighboring values in the circle; [34]). Nevertheless, we were not interested in the exact position of the centroid, but rather the direction of the centroid from the center.

As can be seen in Fig. 1, the line of the reference category (i.e., parent-childhood proximity) only shows minor deviations from the null-line, which results in a centroid that is almost exactly presented in the middle of the circle. Centroids for parent-child as well as childhood-now deviate from the center away from the higher order value of Conservation towards the higher order value of Openness to change. Interpreted more globally, both centroids head towards the higher order value of growth and anxiety-freedom away from self-protection and anxiety-avoidance (see Fig. 1). This effect is mainly driven by the lower importance of the self-protection values (Conformity-rules, Tradition, and Security-social). Furthermore, the lines for parent-child and childhood-now proximity have a very similar line structure, with only one descriptively larger deviation at the Hedonism value. This speaks to the robustness of the results found for parent-child proximity [28].

## Discussion

The present sample was from German-speaking countries of Europe with a relatively high standard of living. Therefore, we expected that people would be motivated by values associated with growth and anxiety-freedom, rather than self-protection and anxiety-avoidance because the study took place in politically stable, economically-developed countries with a high standard of living where people are generally motivated by an optimistic outlook in life, rather than a pessimistic one (e.g., deficiency model, see [45]). This is what we found in the present work. More precisely, participants who preferred universalism (e.g., commitment to equality, justice, and protection of people) and self-direction values (e.g., freedom to follow one’s ideas and determine one’s own actions), but at the same time showed a lower preference for conformity, tradition, and security values, moved further away than participants with opposite preferences. This pattern was stable across different proximity measures – the distance between participants’ and their parents’ current place of living (i.e., parent-child proximity), as well as the distance between participants’ current place of living and their place of childhood (i.e., place where they grew up: childhood-now proximity). Although we argued that the second distance should be considered more valid than the classical parent-child proximity because not confounded by the moving behavior of parents, both distances basically showed the same pattern. This not only speaks to the robustness of our results, but suggests that the moving behavior of parents is probably unsystematic, i.e., parents do not seem to systematically move towards or away from their children. This is underlined by the similar median distances of parent-child proximity and childhood-now proximity (38.3 vs. 38.9; see Table 1).

Regarding the typology of potential motivations to move [38], participants in the present study were motivated by the general motives of self-development (personal growth, acquiring new knowledge and skills) and idealism (building a better world). On the contrary, participants who were instead motivated by preservation motivations (physical, social, and psychological security for oneself and one’s family) moved less further away from their parents’ home. Interestingly, materialism (financial well-being, wealth, and control over material resources) did not seem to have an influence on the motivation to move. This also seems reasonable because all German-speaking countries generally have a high standard of living.

In the current study, we also showed that expected weak effects could be assessed with enough statistical power to finally reveal an expected pattern. The overall explained variance was small (~8 %) because of the multi-causal nature of moving behavior, but still in line with past research [21, 37]. If we compare the predictive value of demographics and human values, both parts explained similar *R*^2^ values. Because human values outperformed education as a known strong predictor of moving and moving distance (4.2 vs. 2.7 % and 4.0 vs. 2.2 %, respectively), this speaks to the meaningfulness of measuring basic human values in the context of moving behavior (see also [38, 42]).

### Methodological considerations

To quantify the influence of several predictors on an outcome measure, multiple linear regressions are usually calculated. In the present study, we showed that, when small effects are expected, many predictors are included in the model, and multicolinearity is probable, a dominance analysis is superior. The dominance analysis has the further advantage of giving more weight to the values itself, rather than significance levels. This is an oft-stipulated requirement to improve replicability and reproducibility of psychological research [11, 12].

Furthermore, we wish to discuss the validity of the new 19-value segmentation of basic human values, which was recently introduced by Schwartz et al. [34]. This new segmentation has two new values (Humility, Face) and six values are now more differentiated (Universalism, Benevolence, Conformity, Security, Power, and Self-direction) compared to the old model [31]. Vecchione et al. [42] have already shown that, at least for the security value, the segmentation into ‘Security-personal’ and ‘Security-social’ is meaningful. In the current study, we found the strongest support for the new segmentation for the Conformity value. People who moved further away from their parents or childhood place judged the importance of complying with rules, laws, and formal obligations (i.e., reflected in the Conformity-rules value) as less important than people who stayed rather close to their parents and childhood place. However, this pattern was not found for the Conformity-interpersonal value. The moving distance was not substantially associated with avoiding behaviors that could upset or harm other people. Therefore, our results present additional support for the validity of the new 19 value segmentation.

### Limitations

Although values are considered to be relatively stable over time, past research has found that values do change occasionally, and that these changes are systematic and meaningful (e.g., [3]). Therefore, in the present study, we cannot infer whether a certain constellation of value-importance is the cause or the consequence of a larger distance between parents and (adult) children. It could also be that the uncovered constellation is the result of fitting into a new life situation after moving to a new place by adjusting to the new life situation (e.g., [40]). Nevertheless, we do think that value preference changes due to changing one’s residence are probably not that substantial in the present study.

The moving behavior observed almost entirely reflected migration within the same cultural area. Participants did not have to adjust their values system as much as, for example, individuals moving to a different culture with a different language, customs, traditions, or religion. Furthermore, because values are usually relatively stable over time (i.e., rather traits than states), it seems plausible that some values become more important or less important, but this change is not so strong as to result in a switch of value preferences to reluctance. Although we cannot entirely settle this issue with the current study because of the cross-sectional design, future research would profit from conducting longitudinal studies about motivations to move.

Although the power of the study design was large enough to detect even small effect sizes reliably, one might argue that the reported pattern (see Fig. 1) in fact reflects measurement error. We tried to address this problem by introducing a third proximity measure that should have no connection with participants’ motivation to migrate. We chose the distance between participants’ place of childhood and their parents’ current place of residence (i.e., parent-childhood proximity). This distance should only reflect motivations to move of participants’ parents and not of participants themselves. Indeed, the regression model was not significant, cumulated dominance weights of all human values were low (1.4 %), and the centroid in the circular model (see Fig. 1) was almost exactly in the middle of the circle.

The present study also had a number of methodological limitations. Most importantly, the subscales of the used PVQ-R have only 3 items each. This leads to higher fluctuations in reliability estimates, which was also found in the present study. For some subscales, the reliability was unacceptably low from a methodological point of view (e.g., Humility), although in line with the original publication of the PVQ-19 [34]. Nevertheless, future research could advance the PVQ-R by including further items for each value to substantially raise reliability or at least revise the items of the Humility subscale.

Because the operationalization of distance was established as the distance between postal code areas (due to privacy issues), quite a few participants had distance = 0. This does not necessarily mean that these participants still live with their parents; it only means that participants and their parents share the same postal code area. To account for this, we calculated the regression analyses again by excluding participants with distance = 0. Results only slightly changed, probably due to the lower number of participants (parent-child proximity and childhood-now proximity: regression models still significant with nearly equal explained variances: 7.8 and 7.9 % respectively; control group parent-childhood proximity: model still not significant; explained variance increased to 5.0 %). Due to power considerations, participants with distance = 0 remained in all analyses.

### Future directions

Future studies would profit from applying a longitudinal design to disentangle motivational causes and consequence of moving behavior. Furthermore, it would be of interest to examine whether the found pattern is also be found in different cultures. This would not only add to the generalizability of the effect in other countries, it would also add to the theory about the motivations to (e)migrate. As we have outlined in the Introduction, in industrialized countries people are motivated more by optimistic goals (e.g., individual growth, achievement), whereas in countries with political and/or economic problems, people are motivated more by pessimistic goals (e.g., fear reduction, raise personal security; [45]). If this holds true, then we would expect the centroid from Fig. 1 being in the lower part of the circular model for countries with political turmoil or economic upheavals.

Another interesting point for future research is of a methodological nature. In the current study, we were not only interested in the distance itself, but also the direction of moving might be of value. For example, do parents move towards their children or away from their children, and to what extent? Geographically speaking, what is the angle between the childhood-now proximity line and the parent-childhood proximity line? If this angle is sharp, then parents did move towards their (adult) children’s current place of living. The more obtuse this angle, the less close parents moved towards their children. If this angle is larger than 90°, then parents moved away from their children, and so forth. This additional measure would complement the classical proximity measure, and could add to the understanding of psychological underpinnings of moving behavior.

Further potential predictors of parent-child proximity, which were not analyzed here, would also be of interest. For example the emotional attachment to parents or in general the rearing behavior of parents could have an influence on the parent-child proximity.

### Ethics approval and consent to participate

The present study was conducted in accordance with the principles of the Declaration of Helsinki and with institutional guidelines of the School of Psychology, University of Vienna. Furthermore, the present study followed the Guidelines for ethical conduct of behavioral projects involving human participants proposed by the American Psychological Association. According to the institutional guidelines of the University of Vienna, Austria (http://satzung.univie.ac.at/ethikkommission-der-universitaet-wien/), approval by an ethics committee was not necessary because the study did not affect the physical or psychological integrity, the right for privacy, or other personal rights or interests (see §2(1)). All participants gave verbal informed consent after having received a written description of the study and could withdraw participation at any point. Data collection was anonymous and no harmful procedures were used.

### Consent for publication

Not applicable.

### Availability of data and materials

Because we do not have the consent from the participants to make the data publicly available through open access repositories, the dataset supporting the conclusions of this article is only available from the first author on demand. All relevant materials can be accessed via the Open Science Framework platform (https://osf.io/pcgy7/).

## Footnotes

1 We also applied a different transformation called Rankit, which is also applicable for this type of data. Because log-transformations and Rankit transformations were highly correlated (Pearson correlation *r* > .92), we kept the log-transformation because it is predominantly used in the literature on parent-child proximity.
